# Real-time nanomechanical property modulation as a framework for tunable NEMS

**DOI:** 10.1038/s41467-022-29117-7

**Published:** 2022-03-18

**Authors:** Utku Emre Ali, Gaurav Modi, Ritesh Agarwal, Harish Bhaskaran

**Affiliations:** 1 Department of Materials, University of Oxford, Oxford, OX1 3PH UK; 2 Department of Materials Science and Engineering, University of Pennsylvania, Philadelphia, PA 19104 USA

**Keywords:** Electronic devices, Electrical and electronic engineering

## Abstract

Phase-change materials (PCMs) can switch between amorphous and crystalline states permanently yet reversibly. However, the change in their mechanical properties has largely gone unexploited. The most practical configuration using suspended thin-films suffer from filamentation and melt-quenching. Here, we overcome these limitations using nanowires as active nanoelectromechanical systems (NEMS). We achieve active modulation of the Young’s modulus in GeTe nanowires by exploiting a unique dislocation-based route for amorphization. These nanowire NEMS enable power-free tuning of the resonance frequency over a range of 30%. Furthermore, their high quality factors ($Q$ > 10^4^) are retained after phase transformation. We utilize their intrinsic piezoresistivity with unprecedented gauge factors (up to 1100) to facilitate monolithic integration. Our NEMS demonstrate real-time frequency tuning in a frequency-hopping spread spectrum radio prototype. This work not only opens up an entirely new area of phase-change NEMS but also provides a novel framework for utilizing functional nanowires in active mechanical systems.

## Abstract (web summary)

Direct modulation of Young‟s Modulus to affect mechanical resonances in real-time has not been achieved before. Here, the authors leverage the dislocation migration phenomenon in GeTe nanowires to develop nanoelectromechanical systems with powerfree tuning of mechanical resonances within a range of 30%, high and stable quality and gauge factors.

## Introduction

A century after the invention of the radio, the quest for the ideal electronic tuner is still ongoing^1^. Owing to extremely-low power requirements (less than a picowatt) and ultra-high quality ($Q$) factors^2^ in NEMS resonators, tunable NEMS have the potential to be the ultimate building blocks in communication electronics and signal processors^3^. Currently, tuning of NEMS resonators is achieved by active stress-tuning^4^ based on the continuous application of net forces through electromagnetic fields (capacitive^5,6^, electrothermal^7^, piezoelectric^8^, dielectric^9^, and magnetomotive^10^ tuning). This is analogous to the adjustment of guitar strings using tuning pegs: higher string tension leads to higher resonance frequency (${f}_{0}$). However, all stress-tuning methods are volatile, i.e., the frequency reverts to its initial state when the stress is removed. Moreover, altering the stress of as-fabricated nanomechanical resonators by an external force dramatically modifies their surface states and clamping conditions, which results in their $Q$ factors changing almost an order of magnitude within the tuning range^4,7^. Here, we overcome these limitations by exploiting a unique characteristic of phase-change materials (PCMs)^11^, i.e., the two states of such materials, crystalline and amorphous, have different Young’s modulus ($E$)^12,13^.

Crucially, previous work on single-crystal PCM nanowires has shown that the concept of dislocation pile-up via the carrier wind force contributes to the amorphization process through a non-melting route^14–17^ (compared to the melt-quench process in thin-films which is challenging in NEMS^18^). PCM nanowires are further shown to display lower structural drift that improves reliability and stability^19^, ultra-low thermal conductivity that dramatically reduces switching energy^20^, with a well-understood defect generation/annihilation mechanism that enables controllable tuning of structural properties^14–17^. These advantages are what we make use of in this work to create a tunable NEMS resonator that functions by altering the mechanical properties of the nanowire permanently yet reversibly. Our approach is dissimilar to previous demonstrations of volatile micromechanical tuning via VO_2_ thin-films^21^. In this paper: (1) we utilize an active nanowire NEMS resonator made of germanium telluride (GeTe); (2) we exploit the unique phase transition properties of GeTe nanowires compared to bulk and thin-film PCMs, particularly in dislocation dynamics to create non-volatile tunability; and (3) we demonstrate an intrinsic transduction (piezoresistive) mechanism that enables us to do in situ read-outs.

## Results

### Device characterization

**Fig. 1:**
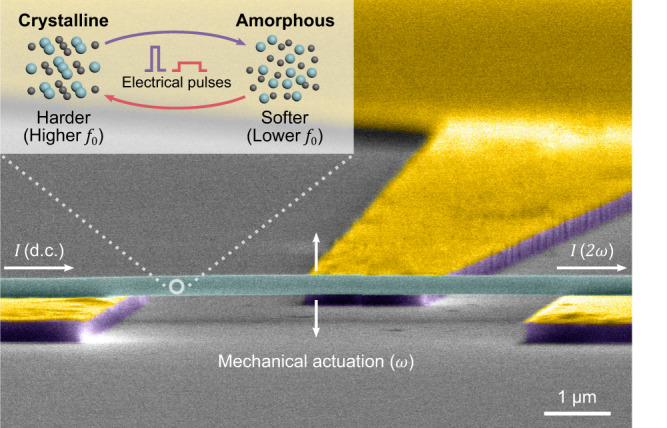
Annotated SEM image of the phase-change NEMS device. The GeTe nanowire with a diameter of ~300 nm is suspended and anchored by Au electrodes. The device is piezoresistively transduced using a d.c. bias source. Symmetrical vibrations of the resonator modulate the d.c. current through the device at a frequency of $2\omega$, doubling the actuation frequency $\omega$. (Inset) The concept of phase-change tuning. Electrical pulses alter the crystallinity of the resonator between a softer amorphous lattice and a harder crystalline lattice, resulting in active tunability of ${f}_{0}$.

An angled-view false-color scanning electron microscopy (SEM) image of the fabricated phase-change NEMS resonator is given in Fig. 1 (refer to Supplementary Note 1 for fabrication details). Nanomechanical measurements of our GeTe nanowires (Supplementary Fig. 1) were performed using the piezoresistive scheme annotated in Fig. 1 (see Supplementary Note 2 and Supplementary Fig. 2 for the detailed schematic of both transduction and tuning circuitry). Briefly, the nanowire was biased with a low-noise d.c. voltage supply, e.g., a battery. A sinusoidal drive signal was externally applied to the (piezoceramic) actuator in order to drive the nanowire into resonance through direct mechanical coupling. At resonance, vibrations of the nanowire alter its d.c. resistance ($R$) owing to piezoresistive effect, modulating the current flow. This current variation was then sensed by a lock-in system to obtain the frequency response of the resonator. Resonance tuning of the device was actively achieved by sending electrical pulses through the nanowire, which in turn softens the resonator through amorphization or hardens it through crystallization. All measurements were carried out at room temperature and in a vacuum environment (~ 10^−6^ mbar).

Mechanical actuation at a frequency of $\omega$ generates a piezoresistive current at $2\omega$ (Fig. 1). Such frequency doubling behavior of self-sensing nanowire resonators has been previously discussed by He et al.^22^, where the relative resistance change due to the (symmetric) elongation of the nanowire is given as:

1 $$\frac{\triangle R}{R}\approx 2.44{\left(\frac{{d}_{{{{{{\rm{c}}}}}}}}{L}\right)}^{2}\gamma$$

Here, $\triangle R$ is the absolute resistance variation due to mechanical motion, ${d}_{{{{{{\rm{c}}}}}}}$ is the displacement at the center of the nanowire while $\gamma$ and $L$ are the gauge factor and the length of the nanowire at rest, respectively. Due to this quadratic dependence of resistance on displacement, frequency doubling takes place. Our measurement technique, which we term true piezoresistive sensing, further reduces the complexity of the setup as we only require a d.c. battery without the need for complicated mixing techniques^22^. Thus, the electrical readout signal $\left|S\left(\omega \right)\right|$ that yields the magnitude response of the resonator becomes a Lorentzian line with a linear background. For our case, i.e., including the frequency doubling effect, $\left|S\left(\omega \right)\right|$ can be analytically approximated as

2 $$\left|S\left(\omega \right)\right|=A+B\omega +\frac{K}{{Q}^{2}}\frac{{{\cos }}\left(\triangle \varphi +2{{{\tan }}}^{-1}\left(\frac{{\omega }_{0}\omega /Q}{{{\omega }_{0}^{2}-\omega }^{2}}\right)\right)}{{\left(1-{\left(\frac{\omega }{{\omega }_{0}}\right)}^{2}\right)}^{2}+{\left(\frac{\omega /{\omega }_{0}}{Q}\right)}^{2}}$$

where $A$ and $B$ are the fitting parameters for the electrical background, $K$ is the peak height, $\triangle \varphi$ is the phase delay between electrical and mechanical domains, $Q$ is the quality factor, and ${\omega }_{0}=2\pi {f}_{0}$ is the resonance frequency. For a detailed derivation of $\left|S\left(\omega \right)\right|$, see Supplementary Note 3 and Supplementary Fig. 3.

**Fig. 2:**
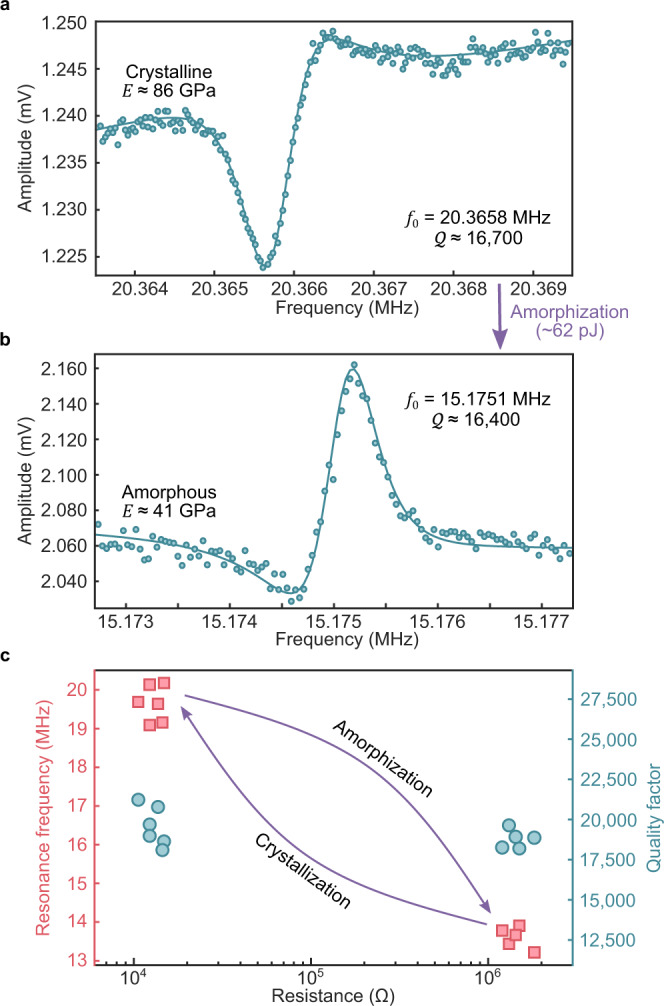
Electrical testing and tuning of the phase-change nanowire. **a** Frequency response of a 7 μm-long virgin nanowire ($R$ = 1.15 kΩ). The plot shows the raw measurement data (dots, 10 averages, 100 Hz bandwidth) for a drive voltage ${V}_{{{{{{\rm{drive}}}}}}}$ = 450 mV. The Lorentzian fit with a linear background (solid line) yields ${f}_{{{{{\rm{cry}}}}}}$ = 20.3658 MHz and $Q$ ≈ 16,700. Young’s modulus in the crystalline state is ${{E}}_{{{{{{\rm{cry}}}}}}}$ ≈ 86 GPa. **b** Raw data (dots, 10 averages, 100 Hz bandwidth) of the amorphized nanowire ($R$ = 0.16 MΩ) after a 1.2 V–50 ns electrical pulse, for the same drive level. The fit (solid line) suggests the resonance frequency is tuned down to ${f}_{{{{{{\rm{amo}}}}}}}$ = 15.1751 MHz while the quality factor remains almost the same ($Q$ ≈ 16,400). The effective Young’s modulus in an amorphous state is estimated as ${{E}}_{{{{{{\rm{amo}}}}}}}$ ≈ 41 GPa. **c** Cyclability test of another 7 μm-long nanowires. The scatter plot shows the resonance frequency (red squares, left-axis) and the quality factor (green circles, right-axis) of another 7 μm-long nanowires versus its electrical resistance for many tuning cycles (3.5 V–50 ns pulses for amorphization and Joule annealing at 1 μA for crystallization). The mean resonance frequency ratio between two phases is ${\bar{f}}_{{{{{{\rm{cry}}}}}}}/{\bar{f}}_{{{{{{\rm{amo}}}}}}}$ = 1.45, and the $Q$ (19,210 ± 1034) shows no dependence on crystallinity.

Initially, the d.c. bias voltage across the nanowire was set to 10 mV, with its electrical resistance measuring as 1.15 kΩ. Figure 2a shows the raw experimental data for the fundamental mode of the virgin GeTe nanowire (length ($L$) = 7 µm; diameter ($D$) = 300 nm; from Fig. 1). The Lorentzian fit (Eq. (2)) to the resonance peak yields a center frequency ${f}_{0}$ = 20.3658 MHz and a quality factor $Q$ ≈ 16,700 with $Q$ × ${f}_{0}$ ≈ 3.4 × 10^11^ Hz. This $Q$ value is of the same order or higher than previously reported high-$Q$ nanowire NEMS resonators at room temperature^23–25^ and comparable to those of time-keeping MEMS devices^26^. We mainly attribute this to our fully mechanical nanowire pick-and-place process as well as the fabrication quality of our pristine nanowires.

In Fig. 2a, the background in the measurement stems from the second-order harmonic distortion of the driving signal capacitively coupling into the readout node. One can eliminate this background by implementing mixing techniques^22^ as previously mentioned and/or minimise it by taking a differential readout^27^. Further, we have obtained the data in Fig. 2a, b with a driving signal amplitude ${V}_{{{{{{\rm{drive}}}}}}}$ = 450 mV, which roughly translates into a driving power level in the picowatt range. We estimate this power level by measuring the effective surface vibrations transferred to the device from the piezoceramic actuator, and the details can be found in Supplementary Fig. 4.

With a density of 6060 kg m^−3^ in crystalline phase^28^, the measured Young’s modulus (${E}_{{{{{{\rm{cry}}}}}}}$ ≈ 86 GPa) is in good agreement with previously reported values^29^. The piezoresistive gauge factor of the crystalline nanowire is calculated to be ${\gamma }_{{{{{{\rm{cry}}}}}}}$ = 19.3 (Eq. (1), Supplementary Fig. 5, and Eq. (S9)), which is higher than those of metallic thin films^30^. This is in line with the fact that GeTe has *p*-type metallicity in the crystalline phase due to the high concentration of intrinsic Ge vacancies^16^.

### Phase-change tuning of nanomechanical resonance

After the amorphization of the nanowire with a ~62 pJ electrical pulse (1.2 V – 50 ns), its d.c. resistance rises to 0.16 MΩ. The resulting resonance peak is given in Fig. 2b. The most surprising finding here is that the resonance frequency is tuned by 26% down to ${f}_{0}$ = 15.1751 MHz. We assume the phase transformation does not alter the mass and the length of the device^31^. For a density value of 5600 kg m^−3^ in amorphous phase^28^ – and including geometric variations, this frequency shift corresponds to an effective softening in Young’s modulus for ~52% (${E}_{{{{{{\rm{amo}}}}}}}$ ≈ 41 GPa). Although a similar amount of softening in phase-change materials has been observed with PCM-coated Si cantilevers before, the frequency shift was <1% as the PCM thin film constituted only a small fraction of the total device volume (PCM:Si = 1:15)^31^. More crucially, no power is required after the tuning event because of the non-volatile nature of the phase-change process, which makes this device particularly suitable for power-sensitive applications such as mobile and satellite communications. We have also found that the gauge factor in the amorphous phase rises to ${\gamma }_{{{{{{\rm{amo}}}}}}}$ = 1100, showing more than a 2-fold increase in the previously reported values for amorphous phase-change thin films (~338)^32^ and is comparable to that of Si nanowires (~2000)^33^. Such an enhancement in piezoresistive effect can be explained by the increased surface-to-volume ratio of the nanowires compared to thin films^33^ and the metal-to-insulator transition of the material via amorphization, respectively^16^.

Another remarkable observation from Fig. 2b is that the $Q$-factor in the amorphous state ($Q$ ≈ 16,400) is almost unchanged from the crystalline state. It is worth noting that in PCM nanowires, the amorphization process does not start at the surface via melting but with the condensation of Ge vacancies along $\left\{111\right\}$ planes throughout the crystal^16,17^. This is in contrast to the random formation of amorphous domes in PCM thin films via melt-quenching, which causes excessive stress build-up at the surface upon amorphization. In free-standing nanowires, the stress is efficiently relieved owing to their high surface-to-volume ratio^19^. We also know from our previous studies^14,17^ that the phase-change happens in the suspended region, so the acoustic coupling condition to the clamps should not differ between the two states. Given that surface effects and clamping conditions are the most dominant loss mechanisms (loss $\propto {Q}^{-1}$) in NEMS resonators^3^, it can be surmised that phase-change events in nanowires are not likely to degrade the $Q$ factors, as experimentally observed.

To verify the initial findings on tuning range and $Q$ factor, we performed further tests with another device ($L$ = 7 µm, $D$ = 280 nm). The resonance frequency of this device changed from 19.1583 to 13.2158 MHz upon amorphization by a ~ 64 pJ pulse while its $Q$ factor slightly increased from 18,100 to 18,800. For recrystallization, we adopted threshold switching via Joule annealing (~ 1 µA d.c.) to ensure defect homogenization^15^ although pulse durations as low as 250 ns are also sufficient^14^. Thus, we cycled the device between two phases while keeping track of its ${f}_{0}$ and $Q$ values (Fig. 2c). The resonance frequencies measured in the crystalline phase have a mean and a standard deviation of ${f}_{{{{{{\rm{cry}}}}}}}$ = 19.65 ± 0.46 MHz while in the amorphous phase, it becomes ${f}_{{{{{{\rm{amo}}}}}}}$ = 13.60 ± 0.28 MHz, yielding a significant ratio of ${\bar{f}}_{{{{{{\rm{cry}}}}}}}/{\bar{f}}_{{{{{{\rm{amo}}}}}}}$ = 1.45. Given that crystalline-to-amorphous transformation is the most energy-consuming phase transition, the spectral performance of the device can be calculated as ~10.6 aJ/Hz. This is approximately 3× improvement in comparison to the theoretical values calculated for thin-film phase-change resonators^18^, owing to the lower thermal conductivity of nanowires (~3.2 W m^−1^ K^−1^ in GeTe thin films, ~1.4 W m^−1^ K^−1^ in GeTe nanowires)^20,34^. As for the quality factors, we observed only minor changes ($Q$ = 19,210 ± 1034) during the cyclability test, regardless of the crystallinity just as in the previous case, further suggesting the dominance of surface effects in its determination.

### Intermediate frequency states

**Fig. 3:**
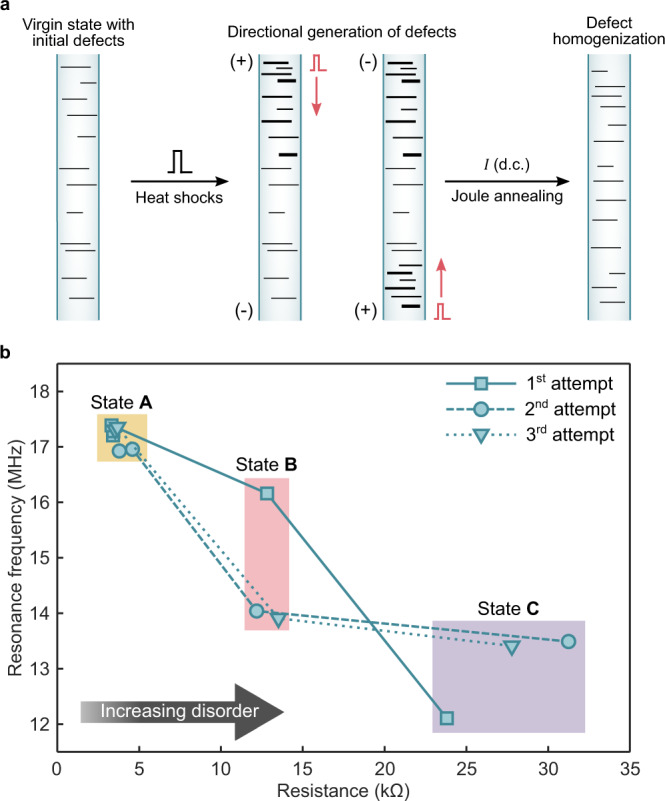
Partial lattice relaxation process in phase-change nanowires via defect engineering and its utilization as a frequency tuning method. **a** The concept of the polarity-dependent, pulse-driven dislocation generation/annihilation mechanism that subsequently relaxes the nanowire lattice. Note that the dislocations are generated near the anode side, making the directional generation of defects possible. To revert the defect concentration to the initial state, Joule annealing is used. **b** The effect of defect generation on the resonance frequency (${f}_{0}$) of a 7 μm-long nanowire. The first few low-energy pulses at the beginning of each attempt result in a marginal shift in the resonance frequency (state **A**) due to further annealing of contacts. Relatively higher-energy pulses are able to increase the lattice disorder, causing ${f}_{0}$ to gradually decrease as seen in the main plot (states **B** and **C**). The distinctive frequency evolution behavior in the first attempt is due to the alternating polarity of pulses (bidirectional pulsing) while the polarity was kept constant during the last two attempts (unidirectional pulsing).

The solid-state, dislocation-based amorphization mechanism via vacancy condensation in single-crystal nanowires also enables accessing multiple intermediate states along the amorphization pathway^14^. We further studied the interesting possibility of obtaining non-volatile intermediate frequency levels. Here, instead of applying 50-ns pulses, which abruptly nucleate the amorphous phase by vigorously accumulating and moving defects, smaller pulse durations (~ 20 ns) were used which can controllably create and accumulate Ge vacancy clusters via heat shocks that prefer to condense in $\left\{111\right\}$ planes (Fig. 3a)^14,16,35^. Provided that the carrier-wind force, i.e., hole-wind force is along the nanowire growth direction $\left\langle 110\right\rangle$, these vacancy loops start to propagate and dissociate with the activation of $\left(111\right)\left[1\bar{1}0\right]$ slip system^16^. At this point, it is crucial to note that the energy levels to activate these slip planes in perfect (defect-free) GeTe have been found to be as low as 340 mJ m^−2^ by density functional theory (DFT) calculations^16^. To achieve a similar mechanism in other covalently-bonded systems such as silicon, the required energy density is higher than 1500 mJ m^−2^, which further proves the unique feasibility of phase-change nanowires for such electrically-driven structural and chemical modification^16,17,35–39^.

As previously demonstrated by the in situ transmission electron microscopy (TEM) studies^15–17^, defects are more likely to be generated near the positive electrode due to the higher momentum of carriers at that side. Hence, by changing the polarity of pulses, bidirectional generation of defects is also possible^17,20^. Moreover, it has been previously shown that annealing via Joule heating can homogenize the defects back to the initial concentration, restoring the crystallinity^14,15^.

To investigate the impact of crystallinity modification on the effective Young’s modulus, we keep track of the resonance frequency of a 7 μm-long nanowire ($D$ = 250 nm) while injecting low-energy pulses (20 ns each) with increasing amplitudes into the device. Also, we simultaneously probe the d.c. resistance of the nanowire as proof of microstructural evolution of defects (as was the case for the aforementioned TEM studies), which further helps avoid uncontrolled amorphization. As such, the resonance frequency and the resistance of the device are recorded whenever a significant change is observed in the d.c. resistance. The corresponding data for this experiment is shown in Fig. 3b, with three distinctive resistance regions: state **A** (<5 kΩ), state **B** (10–15 kΩ), and state **C** (>20 kΩ).

With a bias voltage of 5 mV, the resonance frequency and the resistance of the virgin nanowire were found to be 17.205 MHz and 3.40 kΩ, respectively (beginning of the first attempt). In the first experiment, the amplitude of pulses was gradually increased while alternating the polarity at each step (bidirectional pulsing). The exact pulse sequence for this experiment was +0.1 V, −0.2 V, +0.3 V, −0.4 V, +0.5 V, −0.6 V, +0.7 V, −0.8 V, and +0.9 V. During the first few pulses, only marginal shifts were observed both in the resistance and the resonance frequency (state **A**). This is attributed to the annealing of the electrical contacts, which can nanometrically alter the anchoring of the nanowire on the electrodes. The focus in our studies is on the pronounced change between states **B** and **C** for the sake of clarity. The first substantial increase in resistance (to 12.82 kΩ) was observed with a −0.8 V pulse, yielding a resonance frequency of 16.161 MHz. This change in ${f}_{0}$ can be explained by the increased disorder within the crystal, which softens the nanowire through lattice relaxation just as in the amorphization process. At this point, it is worth noting that the long-range order is still crystalline^16^. The next pulse (+0.9 V) further increased the resistance (to 23.80 kΩ) with the resonance frequency decreasing to 12.108 MHz. At this stage, the device was annealed by passing a current (~5 µA d.c.) through the device in order to annihilate the defects because further pulsing would abruptly nucleate the amorphous phase. After annealing, the nanowire state was restored back to 16.926 MHz and 3.53 kΩ (state **A**).

In our subsequent experiments on the same device, the polarity of pulses was kept the same, with the pulsing sequence from + 0.1 to + 0.7 V with increments of 0.1 V throughout the second and the third experiments. The aim of these experiments was to examine the difference between unidirectional and bidirectional defect generation. The first considerable shift moved the nanowire into the state **B** with a +0.6 V pulse (14.041 MHz and 12.18 kΩ), which was then followed by a +0.7 V pulse (to the state **C**), resulting in an increase in the resistance (31.25 kΩ) and a lowering of the resonance frequency (13.490 MHz). By annealing (~ 5 µA d.c.), the device was reverted once again to state **A**. The device was then brought to the states **B** and **C** after the + 0.6 and + 0.7 V pulses respectively, with the resonance frequency (and the resistance) dropping (increasing) from 13.911 MHz (13.16 kΩ) to 13.412 MHz (27.78 kΩ).

These experiments reveal several important features. One is that annealing can thermally erase the memory of the nanowire, restoring both the resistance and the resonance frequency very close to its virgin state. This substantiates previous findings and further supports the idea of thermal instability of intermediate states at high temperatures^14–16^. However, this does not lead to volatility during normal operation because the intermediate states have stability levels exceeding 3 years at room temperature, which allows non-volatile operation just as in the amorphous phase^14^.

It is interesting to note that the tuning efficiency in Fig. 3b is comparable to the amorphization case in Fig. 2c. Given that the length of the amorphous region remains below ~100 nm in GeTe nanowires^14^, this observation further confirms that the tuning is mostly a result of the phenomenon described in Fig. 3a.

The most striking result to emerge from the data in Fig. 3b is that the first experiment (bidirectional pulsing) leads to a distinct evolution of resonance frequency in comparison with the subsequent two experiments (unidirectional pulsing). However, at the same time, the evolution of resistance is relatively invariant in all three experiments. Our hypothesis was that the resistance is only modified by the number of the added defects whereas the resonance frequency also depends on their distribution along the nanowire. Thus, these experiments provide further insight into the defect generation mechanism to an extent that has not been attainable so far. Briefly, since unidirectional pulses modify the lattice only at the anode side of the nanowire, both the mode shape and the resonance frequency are altered in a more dramatic way than in bidirectional pulsing. Consequently, for a similar level of defect concentration, unidirectional pulsing results in a higher amount of tuning as can be seen in state **B**. The relevant finite-element simulations for such distinct tuning of the fundamental mechanical mode are provided in Supplementary Fig. 6 although we acknowledge that it will be necessary for the future to study higher-order mechanical modes to get more clues about the defect generation mechanisms.

Interestingly, as more pulses are injected into the device, the tuning efficiency of unidirectional pulses gets weaker (see Fig. 3b) for the higher resistance state (state **C**). Given the one-dimensional (1D) carrier flow in nanowires^17^, this effect can be explained by the analytical model of Lighthill and Whitham describing traffic flow on a crowded highway^40^. The model predicts a sudden decrease in the mean velocity of the cars entering a congested region. As the cars leave the congested area, however, they can only gain speed at a much slower rate, and this rate decreases with the increasing concentration of vehicles. In our nanowires, since all the extra defects in state **B** are more uniformly distributed in the case of bidirectional pulsing (reducing the amount of electronic congestion), the carriers from further pulses can propagate deeper into the nanowire and create dislocations more effectively (superior tuning). The upshot of having such control over the evolution of mechanical resonance is the possibility of acquiring infinitely many tuning profiles from a single device by simply adjusting the pulsing pattern, each of which takes only 20 ns. This is potentially enabling unique applications in spread spectrum communications.

### Real-time frequency tuning

Spread spectrum techniques aim to expand the actual bandwidth of radio signals beyond what is required to transmit data while offering numerous advantages ranging from suppressing interference (e.g., in hostile jamming, multiple access, or multipath fading) to facilitating global positioning systems^41^. One way to spread the spectrum of a radio signal is to rapidly change its carrier frequency (${f}_{{{{{{\rm{c}}}}}}}$) within the allocated band by following a pattern that is known to both transmitter and receiver, and this technique is called frequency-hopping spread spectrum (FHSS)^41^. With the increasing demand for connectivity in massive IoT networks based on 5G mobile communications, FHSS has seen a renewed interest although some issues at the device level pertaining to hopping speed, design complexity, and power consumption remain unsolved, e.g., the design of the local oscillator (LO)^42^.

**Fig. 4:**
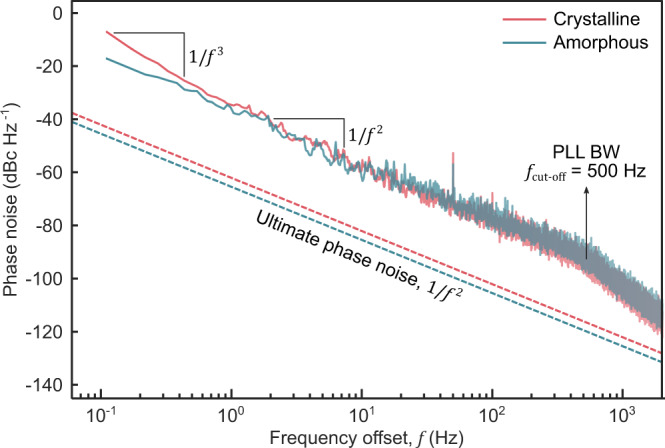
Open-loop phase noise performance of the resonator in crystalline and amorphous states. The measured phase noise data is given by the red solid line in the crystalline state and by the green solid line in an amorphous state, for a carrier signal power of 1.6 nW. The dashed lines of the same colors show the ultimate phase noise performance of the device in corresponding states. This thermomechanical limit can be reached by optimizing the external circuitry.

The key parameter to design a high-fidelity LO is the phase noise performance of the reference frequency source, which, in our case, is the NEMS resonator. Phase noise is a measure of short-term frequency stability and is commonly expressed as “decibels below the carrier per Hertz (dBc Hz^−1^)”^43^. Excessive phase noise causes the LO frequency (${f}_{{{{{{\rm{LO}}}}}}}$) to fluctuate more and interfere with other carriers in the vicinity, leading to an inferior demodulation performance. We quantify the phase noise of our tunable resonator via phase-locked loop (PLL) measurements using the subharmonic detection setting (a built-in feature of the lock-in system) to compensate for the frequency doubling effect (Supplementary Fig. 7). The measured phase noise for a carrier power of ${P}_{{{{{{\rm{c}}}}}}}$ ≈ 1.6 nW in two extreme cases, i.e., crystalline and amorphous states, is given in Fig. 4. The data were obtained for a 7 μm-long nanowire ($D$ ≈ 240 nm), with ${f}_{0}$ = 16.662 MHz ($Q$ = 19,200) in crystalline state and ${f}_{0}$ = 11.190 MHz ($Q$ = 19,000) in amorphous state. In Fig. 4, the solid lines correspond to the experimental data while the dashed lines show the ultimate (thermomechanical) limit to the phase fluctuations in NEMS resonators, as given by the following equation for high-$Q$ systems^3^:

3 $${S}_{\varphi }\left(f\right)=\frac{{k}_{{{{{{\rm{B}}}}}}}T}{4{P}_{{{{{{\rm{c}}}}}}}{Q}^{2}}{\left(\frac{{f}_{{{{{{\rm{c}}}}}}}}{f}\right)}^{2}$$

Here, ${k}_{{{{{{\rm{B}}}}}}}$ is the Boltzmann constant, T is the temperature, and $f$ is the offset frequency from the carrier ${f}_{{{{{{\rm{c}}}}}}}$ ($={f}_{0}$).

Overall, the white noise sources in the experiment dominate the phase noise behavior of the resonator, yielding a headroom of about 30 dB for improvement. Here, the thermomechanical limit can be reached by improved noise matching of the device with the input amplifier and the driving circuitry^43^. Closer to the carrier frequency, the phase noise starts to show $1/{f}^{3}$ dependency in the crystalline phase, which is caused by additional flicker noise ($1/f$) at low frequencies^43^. Given that the flicker noise is associated with the d.c. current (${I}_{{{{{{\rm{noise}}}}}}}\propto {{I}_{{{{{{\rm{DC}}}}}}}}^{0.5}$)^44^, it is expected for the crystalline phase to exhibit poorer flicker noise performance under the same bias voltage as seen from the data. The spurious component at 50 Hz is due to the power line noise and can be eliminated by using fully battery-powered parts in the experiment. The knee at higher frequencies stems from the limited PLL bandwidth (PLL BW) set by the loop filter with a cut-off frequency ${f}_{{{{{{\rm{cut}}}}}}-{{{{{\rm{off}}}}}}}$ = 500 Hz. Note from Eq. (3), the ultimate phase noise attainable from the resonator is better than −125 dBc Hz^−1^ at 1 kHz offset for carrier power levels as low as 1.6 nW. This compares favorably to quartz crystal oscillators while showing at least 6 orders of magnitude improvement in terms of the required carrier power and also satisfying stringent specifications of mobile communications^26,45^.

**Fig. 5:**
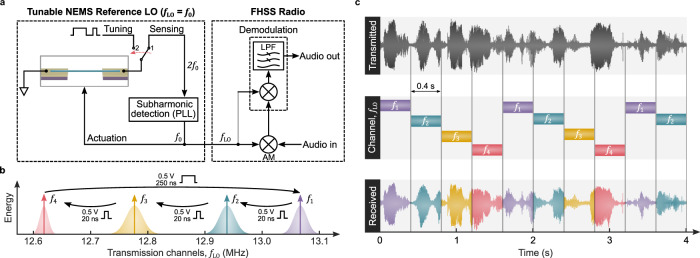
Application idea for the phase-change NEMS resonator as a tunable local oscillator in FHSS systems and the corresponding experimental data. **a** Simplified block diagram of the FHSS radio transceiver exploiting the tunable NEMS resonator as an LO. Subharmonic detection in the LO loop compensates for the frequency doubling effect that stems from self-sensing piezoresistive detection. The RF switch isolates the tuning and electrical sensing circuitry. **b** Channel statistics for the frequency hopping sequence during multilevel operation. The channel frequency (${f}_{{{{{\rm{LO}}}}}}$) was gradually decreased by sending 20 ns–500 mV pulses into the nanowire and recovered via annealing pulses (250 ns–500 mV), which resulted in the following frequency sequence: ${f}_{1}$ = 13.067 ± 0.011 MHz, ${f}_{2}$ = 12.938 ± 0.013 MHz, ${f}_{3}$ = 12.777 ± 0.014 MHz, and ${f}_{4}$ = 12.624 ± 0.006 MHz. **c** Transmitted and received audio segments of 4 s from Guglielmo Marconi’s speech in 1935, together with the real-time frequency hopping sequence (Supplementary Movie 1). Note that each channel is allocated to transmit for only 0.4 s.

In an attempt to demonstrate the idea of phase-change tuning in FHSS systems, we designed an LO utilizing the resonator ($L$ = 8 µm, $D$ = 250 nm) as a tunable reference source, i.e., ${f}_{{{{{{\rm{LO}}}}}}}={f}_{0}$ (Fig. 5a and Supplementary Fig. 7). Here, the RF switch stays in the state “1” by default for frequency tracking (via subharmonic detection) and it is transiently brought to state “2” for tuning events. As the method of broadcasting, we selected amplitude modulation (AM) due to the working frequencies, e.g., ${f}_{0}$ = 13.083 MHz in the virgin state. Prior to the experiment, the d.c. bias current through the nanowire was switched off. The oscillator, as expected, lost track of the reference frequency, further validating the nanomechanical resonance.

In order to hop the transmission channel (or to tune ${f}_{{{{{{\rm{LO}}}}}}}$), electrical pulses (20 ns–500 mV pulses for gradual softening via lattice relaxation and 250 ns–500 mV annealing pulses for hardening) were applied to the nanowire. The period between two tuning pulses was set to 0.4 s, and the LO faithfully tracked the resonance frequency of the NEMS device. We recorded the LO frequency in real-time during the tuning cycles (see Supplementary Fig. 8 for the transient response). The characterization results of 16 frequency levels are given in Fig. 5b, yielding 4 individual channels (${f}_{1-4}$). Here, we observed a maximum frequency deviation of ±14 kHz for channel ${f}_{3}$. As shown in Fig. 5c, the first 10 channels were used as the LO frequency in the FHSS radio for transmitting an audio segment.

We chose a 4 s segment of Guglielmo Marconi’s speech given on 14 December 1930 (*SA 27/9/1 Side B by Essex Record Office*) for this transmission. The audio signal was up-converted (AM) by the hopping sequence using a mixer, and coherent demodulation of the RF signal was performed by the lock-in amplifier. The received waveform was digitally recorded as an audio file and is provided in Supplementary Movie 1. As this data shows, we were able to reliably “switch” the LO frequency, allowing us to transmit this segment of audio through four distinct channels, dynamically changing the channel via switching the nanowire.

This experiment is a demonstration of the applicability of our NEMS device as a tunable LO in FHSS systems. Traditionally, tunable oscillators (i.e., frequency synthesizers) employ non-tunable reference sources, e.g., quartz crystals, due to stability reasons. Integer/non-integer multiples of that reference frequency are then generated within the feedback loop of the LO via frequency dividers. While adding to the overall complexity and power consumption of the system, frequency division operation dramatically impairs the phase noise and the tuning speed^46,47^. The LO topology described here does not need frequency division operation as the reference source itself has non-volatile tunability with excellent phase noise stability and non-changing $Q$ factors (for further discussion on non-changing $Q$ factors, please see Supplementary Note 9 and Supplementary Fig. 9). It is worth mentioning that to perform real-world applications, future research is required to identify effective methods for amplifying the NEMS readout signals, i.e., from nW levels to transmittable mW levels.

## Discussion

In conclusion, we demonstrate a continuous tuning technique for NEMS resonators that actively modulates Young’s modulus of GeTe nanowires. This occurs only in nanowire devices where the dislocation movements are confined, as previously shown. We show that nanosecond-fast electrical pulses can transform the atomic structure, which leads to either softening via amorphization (50 ns) or hardening via crystallization (250 ns), thus yielding a frequency tuning range over 30%. Maintaining the resonance frequency requires no additional stimulus as the phase-change is non-volatile, i.e., it does not change until it is switched again by another pulse, which is a significant advance over devices that use volatile phase change materials such as VO_2_. Multi-frequency operation is also demonstrated through the gradual relaxation of the lattice via 20-ns electrical pulse-induced heat shocks. Unlike traditional stress-tuning schemes, such structural modification does not compromise the high $Q$ factors of our devices (>1.6 × 10^4^), which enables excellent phase noise performance at any frequency state with only picowatt-level driving. The giant piezoresistive gauge factors of these nanowires (${\gamma }_{{{{{{\rm{cry}}}}}}}$ = 19.3 and ${\gamma }_{{{{{{\rm{amo}}}}}}}$ = 1100) are further shown to facilitate a complete integration of the device through self-sensing of nanomechanical resonance. Further, our work offers a cutting-edge solution in wireless and mobile communications by eliminating the need for frequency dividers, which could lead to the simultaneous reduction of tuning speed, power consumption, overall cost, and system complexity. If cyclability and repeatability of these devices (currently ~20 cycles) are enhanced to meet the commercial standards, phase-change nanowire NEMS can serve as the ultimate tunable nanoelectromechanical frequency synthesizers and filters for the future of (massive) IoT and 5G networks.

## Supplementary information

Supplementary Information

Description of Additional Supplementary Files

Supplementary Movie 1

## Data Availability

All the data needed to evaluate the findings of this work are present in the paper and the Supplementary Information. Additional data are available from the authors upon reasonable request.
