# Supplementary material for: Real-time nanomechanical property modulation as a framework for tunable NEMS
Source: Nat Commun. 2022 Mar 18;13:1464. doi: 10.1038/s41467-022-29117-7 (PMC8933423; doi:10.1038/s41467-022-29117-7)
Supplement: Supplementary file 1 — Supplementary Information [file 41467_2022_29117_MOESM1_ESM.pdf]

Supplementary Information for

Real-time nanomechanical property modulation as a framework for tunable NEMS

*Utku Emre Ali, Gaurav Modi, Ritesh Agarwal, Harish Bhaskaran**

*Correspondence e-mail: harish.bhaskaran@materials.ox.ac.uk

This file contains:

- Supplementary Notes 1-9
- Supplementary Figs. 1-9
- References

1 Device fabrication

Fabrication of the electrodes: To manufacture the electrical platform for the testing of free-standing GeTe nanowires, we used a 10×10 mm^2 piece diced from a Si wafer with a thin layer of Si_3N_4 (0.33 µm) on top of a 3 µm-thick oxide layer. The Au electrodes (95 nm) were patterned using electron beam lithography (JEOL 5500FS) and deposited by thermal evaporation with a 5 nm of Cr adhesion layer. With the electrodes acting as a hard-mask, the Si_3N_4 thin-film was anisotropically etched by reactive ion etching (RIE, Oxford 80 Plasmalab). Thus, we formed trenches deeper than 0.4 µm in total by fully removing the Si_3N_4 layer.

Growth of GeTe nanowires: Germanium telluride (GeTe) nanowires were synthesized by a catalyst-assisted vapor-liquid-solid (VLS) growth mechanism[1,2]. As the catalyst, a thin film of Au-Pd (~5 nm) was sputtered on silicon substrate. The synthesis was carried out in a 1-inch tube furnace (Lindberg/Blue M) where a few milligrams of GeTe powder (Alfa Aesar, 99.999% purity) was placed in the centre while the substrate was placed at ~5 inches upstream from the centre. Before heating up the furnace, Ar gas was purged into the chamber several times to remove any residual oxygen until reaching a pressure of 30 mTorr. The base pressure in the furnace was then stabilized at 100 Torr while keeping the argon flow rate at 100 sccm. Subsequently, the temperature of the furnace was ramped up to 650 ℃ and the nanowire synthesis was carried out for an hour. After completion of the growth, the furnace was gradually cooled down to room temperature. The scanning electron microscope (SEM) image of the fabricated nanowires is given in Fig. S1**.**

Deposition of the nanowires over the electrodes: We mechanically transferred the nanowires over the electrodes using a bottom-up approach by utilising polydimethylsiloxane (PDMS) stamps. The steps regarding the method can be found elsewhere[3].

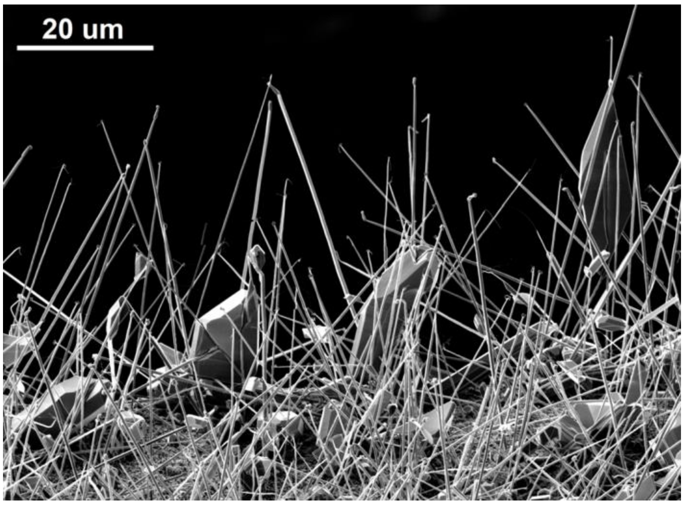

Fig. S1 SEM image of the GeTe nanowires produced by the catalyst-assisted VLS method. The diameters of the rhombohedral ($R3m$) nanowires vary between 150 – 300 nm.

2 Experimental setup for resonance testing and tuning

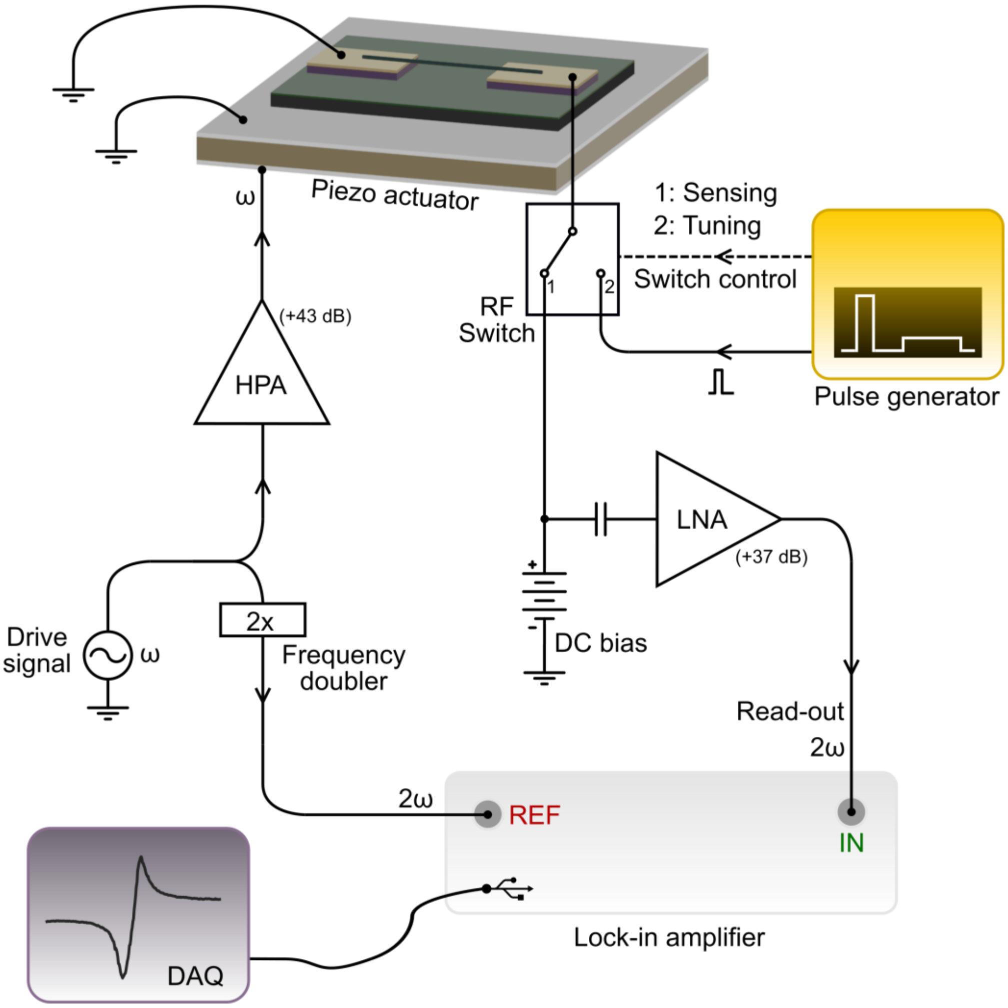

Fig. S2 Electrical measurement and tuning setup. The lock-in amplifier drives the piezo actuator at a frequency of ω and detects the frequency response of the resonator at 2ω reference. Amplification of the drive and the read-out signals is performed by the HPA and the LNA, respectively. The pulse generator is utilised for both tuning the resonator and controlling the RF switch that efficiently isolates the detection and the tuning circuitry.

The setup combining both piezoresistive detection and electrical tuning of the resonator is depicted in Fig. S2. Actuation of the device is performed through direct mechanical coupling using a piezoceramic shaker (~780 pF). The chip was electrically isolated and mounted on the actuator. The drive signal (ω) was sourced by the lock-in system (Zurich Instruments HF2LI) and amplified by the high-power amplifier (HPA, Mini Circuits LZY-22+). Note that the drive signal is applied to the bottom plate of the piezo crystal while its top plate is grounded in order to avoid any performance degradation in the NEMS resonator through capacitive coupling.

For the piezoresistive detection, the d.c. bias voltage across the nanowire is supplied by a battery. When the simultaneous monitoring of the electrical resistance is of interest, e.g. in Figs. 2C and 3B of the main article, the d.c. bias was applied by the source measure unit (SMU, Keithley 2410 SourceMeter). The read-out signal (2ω) was coupled into the low-noise

amplifier (LNA, MITEQ AU-1442) using a coupling capacitor (Crystek DC Block 300 kHz – 3 GHz). The amplified signal was routed into the lock-in amplifier with its demodulation reference set to 2ω.

The tuning of the device was performed by electrical pulses, which were generated by the arbitrary function generator (AFG, Tektronix AFG3151C). It is important to mention that the RF switch (Mini Circuits ZFSWA-2-46) isolates the detection and the tuning circuitry for energy efficiency and is controlled by another AFG (Tektronix AFG3102C). When the switch is in sate '1', the piezoresistive detection is activated. For the electrical tuning, the switch is transiently brought to state '2' connecting the AFG directly to the nanowire. Finally, frequency response of the resonator was acquired by the data acquisition (DAQ) software of the lock-in amplifier through universal serial bus (USB) interface.

3 Non-zero phase Lorentzian fitting of resonance peaks

The motion of a damped harmonic oscillator, e.g. spring-mass system, driven by a sinusoidal force is governed by the following differential equation

$$m\frac{d^2x}{dt^2} + \beta\frac{dx}{dt} + kx = F_0\cos(\omega t) \qquad \text{(S1)}$$

where x is the displacement, m is the mass, β is the linear damping coefficient, k is the spring constant while F_0 and ω are the amplitude and the angular frequency of the driving force, respectively. In steady sinusoidal state, the solution to equation (S1) can be written as

$$x = |x_0|\cos(\omega t + \varphi_0) \qquad \text{(S2)}$$

where the frequency dependent amplitude $|x_0|$ and phase φ_0 of the displacement are:

$$|x_0| = \frac{F_0/m}{\sqrt{(\omega_0^2 - \omega^2)^2 + (\omega_0\omega/Q)^2}} \qquad \text{(S3a)}$$

$$\varphi_0 = -\tan^{-1}\left(\frac{\omega_0\omega/Q}{\omega_0^2 - \omega^2}\right) \qquad \text{(S3b)}$$

Here, $Q = m\omega_0/\beta$ is the quality factor and $\omega_0 = \sqrt{k/m}$ is the resonance frequency of the mechanical system for $\beta^2 \ll 4mk$. As discussed in the main article, the piezoresistive read-out signal $S_{\mathrm{pz}}(\omega)$ in our case varies linearly with the square of the displacement (x^2) unlike the traditional piezoresistive transduction schemes. As given in equation (S4), $|S_{\mathrm{pz}}(\omega)|$ follows the Lorentzian lineshape.

$$|S_{\mathrm{pz}}(\omega)| \propto \frac{1}{(\omega_0^2 - \omega^2)^2 + (\omega_0\omega/Q)^2} \qquad \text{(S4)}$$

Apart from $S_{\mathrm{pz}}(\omega)$, there is also a constant and frequency dependent electrical background response of the experimental setup, $S_{\mathrm{bg}}(\omega)$, regardless of the resonance condition. Hence, the overall lock-in read-out $S(\omega)$ is simply the addition of these two physical quantities, i.e. $S(\omega) = S_{\mathrm{bg}}(\omega) + S_{\mathrm{pz}}(\omega)$. However, it should be noted that due to non-zero delays within the experiment, e.g. non-ideal cables and device parasitics, the coupling between mechanical and electrical domains will not happen instantaneously but with an inevitable phase shift $\Delta\varphi$. In that case, to model the overall frequency response of the device, one should add these two terms considering the $\Delta\varphi$. Within a small frequency interval ($Q \gg 1$), $|S_{\mathrm{bg}}(\omega)|$ can be linearized as $A + B\omega$, where A and B are the fitting parameters. By following a similar procedure that was previously described by Sazonova[4], the resulting amplitude response of the overall electrical read-out can be written as:

$$\begin{aligned} |S(\omega)| &= \mathrm{abs}\{|S_{\mathrm{bg}}(\omega)|e^{i\Delta\varphi} + S_{\mathrm{pz}}(\omega)\} \\ &= \mathrm{abs}\{|S_{\mathrm{bg}}(\omega)|e^{i\Delta\varphi} + |x_0|^2 e^{i2\varphi_0}\} \end{aligned} \tag{S5}$$

Here, $\mathrm{abs}\{\cdot\}$ operator is the same as $|\cdot|$ operator, and it denotes the magnitude of the complex quantity. For $|A| \gg K$, where K is the peak height, $|S(\omega)|$ can be further simplified as:

$$|S(\omega)| \approx A + B\omega + \frac{K}{Q^2} \frac{\cos\left(\Delta\varphi + 2\tan^{-1}\left(\frac{\omega_0\omega/Q}{\omega_0^2 - \omega^2}\right)\right)}{\left(1 - \left(\frac{\omega}{\omega_0}\right)^2\right)^2 + \left(\frac{\omega/\omega_0}{Q}\right)^2} \tag{S6}$$

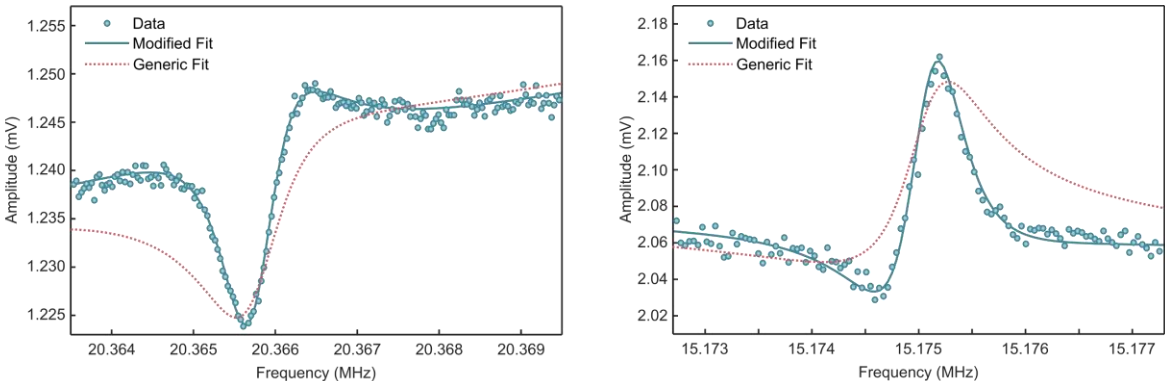

Fig. S3 Frequency response of the resonator and the corresponding fits using the modified model (equation (S6)) and the generic square-root of non-zero phase Lorentzian functions in crystalline (left) and amorphous (right) phases.

In Fig. S3 are depicted two sets of experimental data and best fits to them. As seen, the modified non-zero phase Lorentzian model $|S(\omega)|$ fits well to the data while the generic non-zero phase square root Lorentzian model[4] clearly diverges from the detected signal for the same Q value. This further verifies our assumption of squaring effect made in the main article. The goodness of the fits is quantitated in terms of normalised root-mean-square-error. The

modified model yields much lower error rates (1.1% in Fig. S3(left) and 1.4% Fig. S3(right)) than the generic model, which results in 10.4% and 8.6% in Fig. S3(left) and (right), respectively.

4 Characterisation of the piezo actuator and estimation of NEMS driving power

To calculate the piezoresistive gauge factor of the GeTe nanowire, vibration amplitude of the resonator (a_{NEMS}) needs to be determined for a given drive voltage V_{drive}. In a high-Q NEMS device, a_{NEMS} can be estimated as $Q_{\mathrm{NEMS}} \times a_{\mathrm{piezo}}$, where a_{piezo} is the effective amplitude of surface vibrations of the piezo shaker directly transferred to the nanowire. To this end, we have characterised our piezoceramic actuator using the contact-mode atomic force microscopy (AFM, Asylum MFP-3D) setup depicted in the inset of Fig. S4. Note from the setup that the AFM cantilever is placed on top of the device electrode (not on top of the piezo actuator). Thus, the losses from piezo surface to chip and from chip to devices have been considered in this measurement.

The Lorentzian fit to the resonance curve of the AFM cantilever (Budget Sensors Contact-G) yielded f_{cant} = 11.98 kHz and Q_{cant} = 61. The spring constant of the cantilever was calibrated as 0.188 N m^{-1}, with an inverse optical lever sensitivity (invOLS) of 238.63 nm V^{-1}. Then, we were able to determine a_{piezo} by simply interfacing the lock-in system with the AFM setup (open-loop configuration) such that the lock-in amplifier recorded the detector output (average signal count: 1000) while sweeping V_{drive} (1 – 10 V). The piezo actuator was driven at f_{cant} in order to amplify the detected signal by $\sim Q_{\mathrm{cant}}$. As seen from the data in Fig. S4, a_{piezo} changes linearly with respect to the driving voltage as expected. Thus, the vibration amplitudes for lower V_{drive} levels can be extrapolated from this linear line fit. These results further validate that even with the maximum drive level in this experiment (i.e. 10 V), the piezo actuator stays in linear region.

From Fig. S4, for V_{drive} = 450 mV (and Q = 16,700), the nanowire reaches a maximum displacement amplitude of $a_{\mathrm{NEMS}} \approx 1$ nm. With $\mathcal{E}_0 = m_{\mathrm{eff}} \omega_0^2 {a_{\mathrm{NEMS}}}^2/2$ being the total energy of the resonator during the oscillations, where m_{eff} is the effective mass at the fundamental mode (ω_0), the driving power P_0 required to maintain this vibrational amplitude is calculated as[5]:

$$P_0 = \frac{\omega_0 \mathcal{E}_0}{Q} \quad \text{(S7)}$$

For $m_{\mathrm{eff}} = 1.192 \times 10^{-15}$ kg as estimated by a finite-element analysis software (COMSOL Multiphysics®), equation (S7) yields $P_0 \approx 0.2$ pW, further paving the way for ultralow power applications[6]. For this drive level, the critical amplitude (a_{crit}) for the onset of Duffing nonlinearity is calculated as ~1.76 nm using equation (S8)[7]. Thus, the elastic modulus and

gauge factor calculations in the main article are assumed to be within the linear region of operation.

$$a_{\mathrm{crit}} \approx 0.759 \frac{D}{Q^{0.5}} \tag{S8}$$

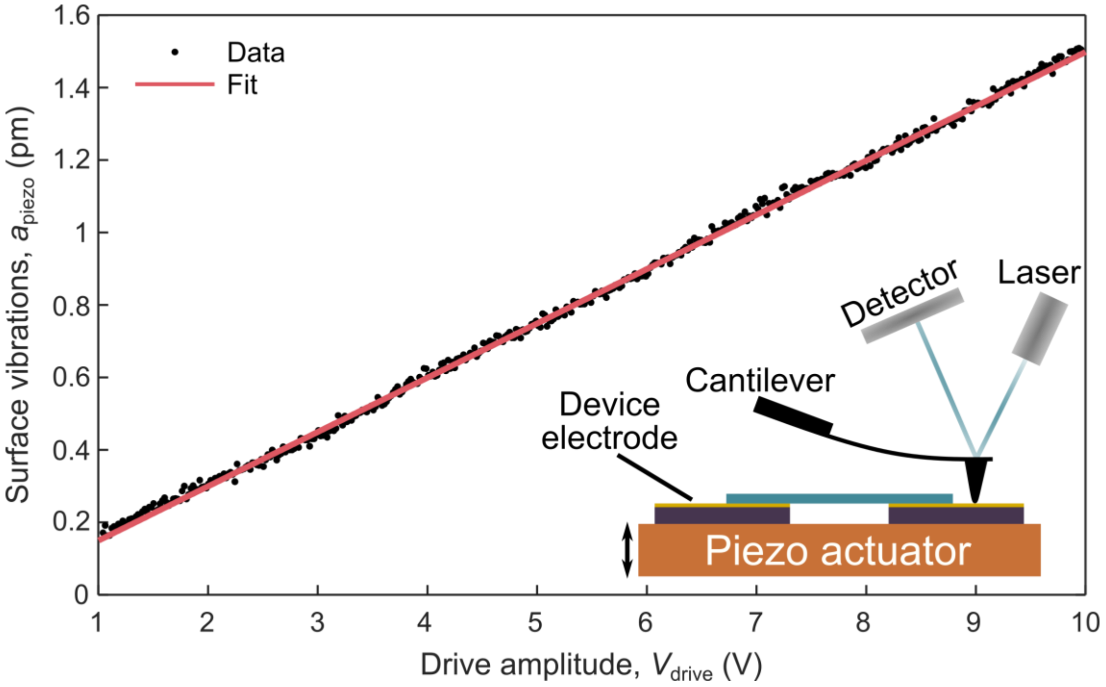

Fig. S4 Characterisation of the piezoceramic actuator using contact-mode AFM. Surface vibrations of the piezoceramic actuator is plotted with respect to the drive voltage. The fit is linear, and the inset shows the schematic diagram of the AFM setup.

5 Electrical equivalent of the experimental setup

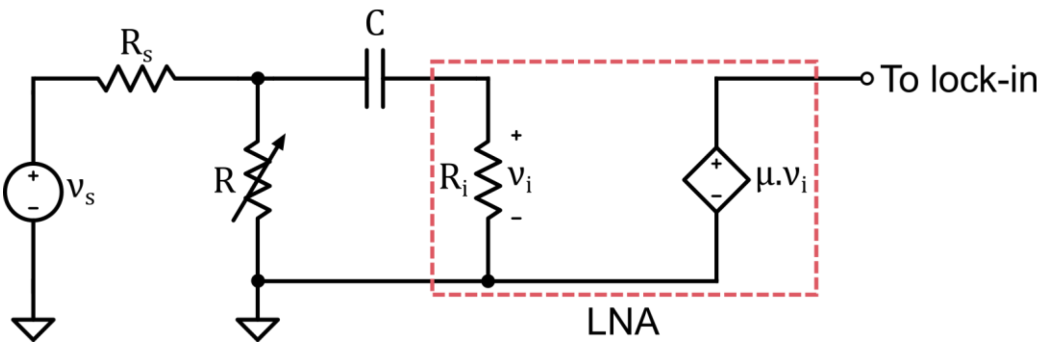

Fig. S5 Schematic of the equivalent piezoresistive detection circuit. The resonator is modelled as a time-varying resistor (R) and the LNA is shown as a VCVS.

In Fig. S5, the electrical equivalent of the piezoresistive measurement setup is given. The d.c. bias source v_s has an internal resistance of R_s. The resistance of the resonator is modelled as a variable resistor $R = R_0 + \Delta R$. Here, R_0 is the d.c. resistance of the nanowire at rest and the time-varying ΔR can be referred to from the Eq. 1 of the main article. The voltage across the

resonator is routed into the LNA. The LNA is depicted as a voltage-controlled-voltage-source (VCVS) with an input resistance of R_i and a gain of μ. The amplified signal ($v_\mathrm{out} = \mu . v_\mathrm{i}$) is subsequently inputted to the lock-in amplifier. Setting $R_0 \gg R_\mathrm{s} = R_\mathrm{i}$ and also assuming C is ideal within the measurement bandwidth, the peak-to-peak amplitude of the output voltage can be written as:

$$\begin{aligned} v_{out,pp} &= \mu v_\mathrm{s} R_\mathrm{i} \left(\frac{1}{R_\mathrm{s} + R_0} - \frac{1}{R_\mathrm{s} + R_0 + \Delta R_\mathrm{max}} \right) 2 \frac{R_\mathrm{s} \parallel R_0}{R_\mathrm{i} + R_\mathrm{s} \parallel R_0} \\ &\approx \mu v_\mathrm{s} R_\mathrm{i} \left(\frac{1}{R_\mathrm{s} + R_0} - \frac{1}{R_\mathrm{s} + R_0 + \Delta R_\mathrm{max}} \right) \end{aligned} \quad \text{(S9)}$$

The expression in equation (S9) was further verified by a general-purpose electrical circuit simulator, e.g. SPICE (Simulation Program with Integrated Circuit Emphasis), via transient analyses using the schematic in Fig. S5.

6 Nanowire tuning behaviour under different pulsing schemes

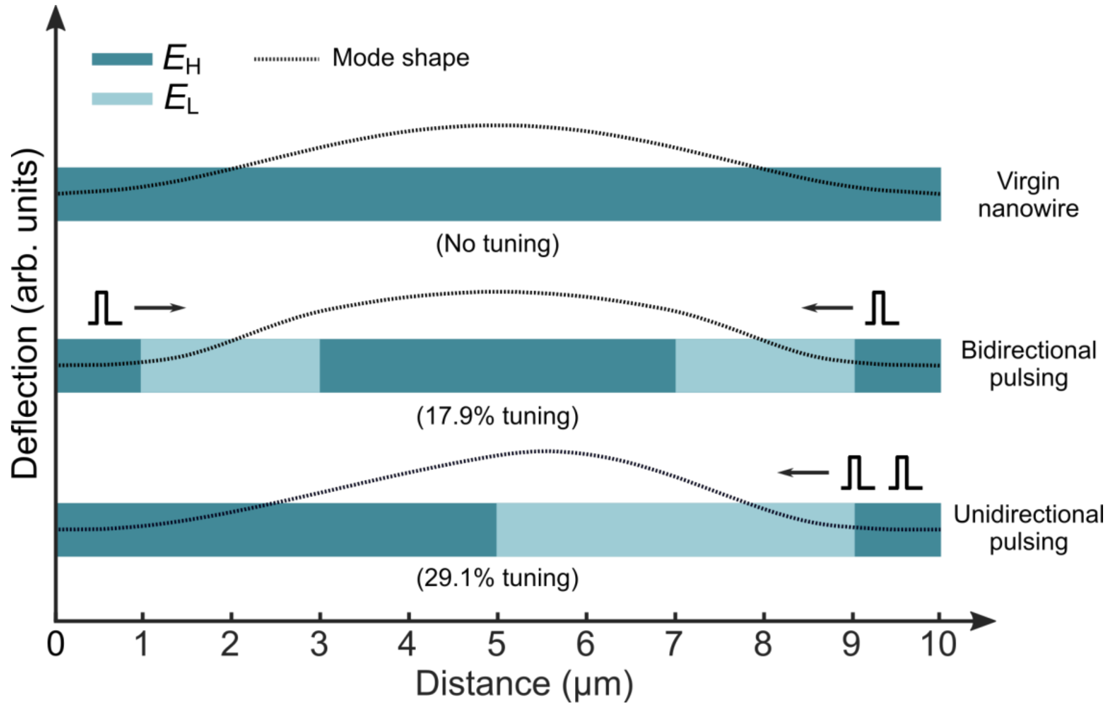

Fig. S6 The effect of directional pulsing on the tuning efficiency and the mode shape. Note that for the same amount of softening, i.e. same length of the softer portion (E_L), unidirectional pulsing yields higher amount of tuning.

To demonstrate the effect of pulsing direction on the tuning efficiency, we have performed finite element analyses (using COMSOL Multiphysics®) on a 10 µm-long nanowire with a diameter of 250 nm. The Young's modulus of the crystalline nanowire (E_H) was set to 86 GPa (see the main article). In both cases, i.e. unidirectional and bidirectional pulsing, the modified portion of the nanowire lattice is assumed to be 4 µm-long in total, with a lower Young's

modulus of E_L = 14 GPa. Here, both values were estimated from the previous literature to yield similar results to our experimental findings[8,9]. Note from Fig. S6 that the softer region is equally partitioned on both sides of the nanowire (2 + 2 µm) in the case of bidirectional pulsing. The virgin nanowire has a resonance frequency of 8.36 MHz. Bidirectional pulsing yields a resonance frequency of 6.86 MHz (17.9% tuning) while unidirectional pulses result in a lower value of 5.92 MHz (29.1% tuning) with a superior tuning efficiency. It can also be seen that the mode shape is dramatically altered in the case of unidirectional pulsing.

7 A note on phase-locked loop (PLL) measurements and the FHSS radio

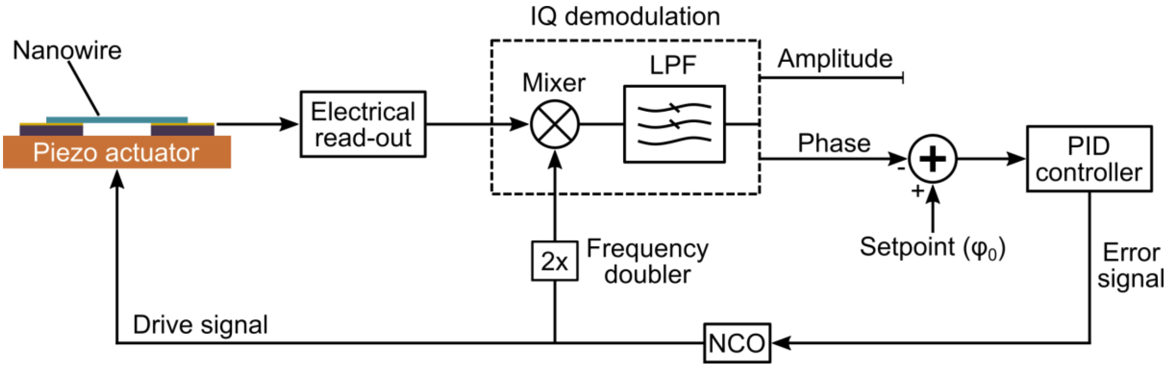

Fig. S7 Interfacing of the NEMS setup with the internal PLL of the lock-in amplifier. The PLL is composed of an IQ demodulator, a PID controller, and a numerically controlled oscillator (NCO). The loop is forced to lock into the subharmonic of the read-out signal to compensate for the frequency-doubling effect caused by the transduction method. This is done via changing the harmonic settings of the phase detector to '2', which logically adds a frequency doubler on the feedback path.

PLLs are based on a negative frequency feedback loop that tries to match the phase and frequency of the loop oscillator with the phase and frequency of the input signal. Fig. S7 depicts the logical block diagram of the internal PLL circuitry within the lock-in system. Here, the loop oscillator is a numerically controlled oscillator (NCO) and the piezoresistive read-out signal is demodulated at the 2nd harmonic of the NCO frequency to compensate for the frequency-doubling effect (subharmonic detection). The PID controller within the loop ensures the phase of the demodulated signal stably matches the setpoint (φ_0).

Note that the NCO output directly drives the resonator in Fig. S7. We use this configuration for the FHSS radio implementation (Fig. 5A), where we form a local oscillator (LO) using the NEMS resonator. However, in the case of phase noise measurements[10] (Fig. 4), we perform open-loop frequency tracking, i.e. drive signal is disconnected from the NCO within the PLL and manually set using a separate internal NCO of the lock-in amplifier. This allows for the true phase characteristics of the NEMS device to be captured[11]. Further we select 500 Hz as the PLL bandwidth during phase noise measurements since the theoretical phase noise of the resonator is better than -110 dBc Hz^{-1} at 500 Hz offset. The relevant PID parameters for the desired loop bandwidth are automatically calculated by the lock-in software. The bandwidth of the low-pass filter (LPF) in IQ demodulator is set to 4 kHz for stability purposes.

For the FHSS radio experiments, the audio signal was produced with a sampling rate of 8 kHz. Up-conversion (i.e. amplitude modulation) of this signal was performed by a double-balanced mixer (Mini-Circuits ZLW-1-1+). For faster locking of the LO, the PLL bandwidth was set to 30 kHz (highest value available from the lock-in system). The cut-off frequency of the lock-in demodulation filter was chosen as 88.39 kHz and the received signal is sampled at 460.53 kHz to prevent aliasing. The demodulated signal was decimated back to its original sampling rate (~8 kHz) and saved as an audio file.

8 Transient response of the LO frequency

Fig. S8 shows the real-time LO frequency tracking data for the experiment in Fig. 5. Here, the sampling period is ~2.17 μs (minimum sampling period attainable from the lock-in amplifier). The data shows the largest frequency tuning step in this experiment, i.e. recrystallization via 250-ns pulse. At point **a**, the RF switch is transiently brought to state ‘2’ for 1 μs for tuning, and a tuning pulse is injected into the nanowire. After 1 μs, the RF switch is brought back to state ‘1’ (back to the sensing/tracking mode). Given that the PLL frequency has already increased at point **b**, it can be surmised that the mechanical resonance frequency is shifted within less than a sampling period. Also note that our main focus in this work is not on the settling time (or lock time) as it strongly depends on the PLL bandwidth, i.e. shorter settling times can be achieved with a larger PLL bandwidth.

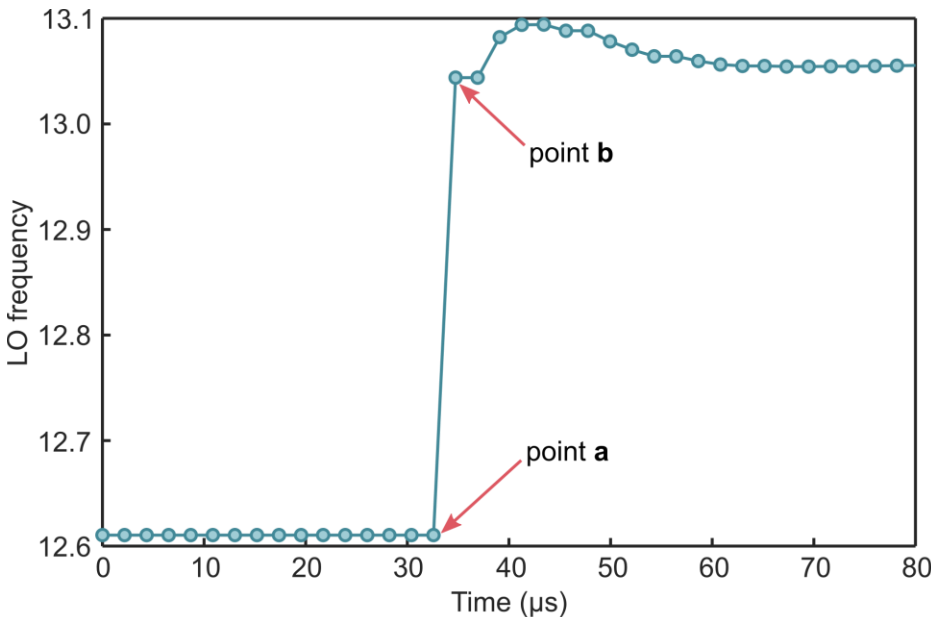

Fig. S8 Real-time LO frequency tracking data for the largest tuning step during the experiment in Fig. 5. The tuning event, i.e. both pulsing and sensing the new frequency, takes place within a sampling period (~2.17 μs).

9 Effect of growth direction on quality factors

The reason why phase-change tuning does not affect the Q factors in this work is actually the non-changing surface states between two phases. Here, the growth direction of these nanowires plays a significant role and causes the defects to propagate along the growth axis, i.e. along $\langle 110 \rangle$ crystalline orientation[12]. This behaviour is dissimilar to other nanowire systems experiencing stress, e.g. the work by Greer and Nix[13], where the defects are pushed towards the surface. Since no new defects (or dangling bonds) are created on the surface, the Q factors remain almost intact.

Fig. S9 shows the SEM image of an eventually burnt-out device. In this experiment, the surface of the nanowire became rougher upon tuning pulses. This dramatic change in the surface states then degraded the Q from ~19,000 to below 500 within eight tuning cycles. This result is a signature of the wrong growth direction, whereby the defects in the nanowire move towards the surface (and not along the growth axis).

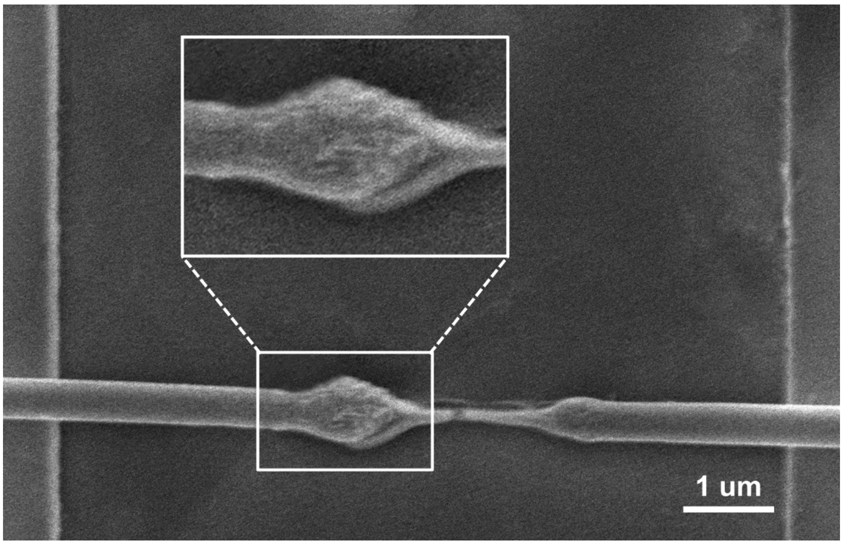

Fig. S9 SEM image of a GeTe nanowire not grown in $\langle 110 \rangle$ direction. The nanowire eventually failed after eight tuning pulses because of the defects propagating towards the surface. This behaviour further increases surface roughness and dramatically impairs the Q factor.

References

1. Nukala, P., Lin, C.-C., Composto, R. & Agarwal, R. Ultralow-power switching via defect engineering in germanium telluride phase-change memory devices. *Nat. Commun.* **7**, 10482 (2016).

2. Lieber, C. M. Nanoscale Science and Technology: Building a Big Future from Small Things. *MRS Bull.* **28**, 486–491 (2003).

3. Lu, Y. *et al.* Mixed-Mode Operation of Hybrid Phase-Change Nanophotonic Circuits. *Nano Lett.* **17**, 150–155 (2017).

4. Sazonova, V. A. A tunable carbon nanotube resonator. (Cornell University, 2006).

5. Cleland, A. N. & Roukes, M. L. Noise processes in nanomechanical resonators. *J. Appl. Phys.* **92**, 2758–2769 (2002).

6. Ekinci, K. L. & Roukes, M. L. Nanoelectromechanical systems. *Rev. Sci. Instrum.* **76**, 061101 (2005).

7. Postma, H. W. Ch., Kozinsky, I., Husain, A. & Roukes, M. L. Dynamic range of nanotube- and nanowire-based electromechanical systems. *Appl. Phys. Lett.* **86**, 223105 (2005).

8. Won, Y., Lee, J., Asheghi, M., Kenny, T. W. & Goodson, K. E. Phase and thickness dependent modulus of $Ge_2Sb_2Te_5$ films down to 25 nm thickness. *Appl. Phys. Lett.* **100**, 161905 (2012).

9. Nam, S.-W. *et al.* Electrical Wind Force–Driven and Dislocation-Templated Amorphization in Phase-Change Nanowires. *Science* **336**, 1561–1566 (2012).

10. Zurich Instruments Newsletter - Edition Q1/2014 | Zurich Instruments. https://www.zhinst.com/europe/en/newsletter/zurich-instruments-newsletter-edition-q1-2014.

11. Roy, S. K., Sauer, V. T. K., Westwood-Bachman, J. N., Venkatasubramanian, A. & Hiebert, W. K. Improving mechanical sensor performance through larger damping. *Science* **360**, (2018).

12. Nukala, P. *et al.* Direct Observation of Metal–Insulator Transition in Single-Crystalline Germanium Telluride Nanowire Memory Devices Prior to Amorphization. *Nano Lett.* **14**, 2201–2209 (2014).

13. Greer, J. R. & Nix, W. D. Size dependence of mechanical properties of gold at the sub-micron scale. *Appl. Phys. A* **80**, 1625–1629 (2005).
