# Supplementary material for: Real-time nanomechanical property modulation as a framework for tunable NEMS
Source: Nat Commun. 2022 Mar 18;13:1464. doi: 10.1038/s41467-022-29117-7 (PMC8933423; doi:10.1038/s41467-022-29117-7)
Supplement: Supplementary file 2 — Description of Additional Supplementary Files [file 41467_2022_29117_MOESM2_ESM.docx]

**Description of Additional Supplementary Files**

**File Name: Supplementary Movie 1
Description:** The movie shows the real-time implementation of our tunable and high-𝒬 NEMS resonator in an AM-based FHSS radio station. In the first part of the movie, the original audio segment (with a duration of 4 s) from Guglielmo Marconi‟s speech given on 14 December 1930 (SA 27/9/1 Side B by Essex Record Office) is being played. In the second part of the movie, the simplified block diagram of the FHSS radio is given together with the transmitted audio signal in the background. The frequency sequence to the radio is generated by the NEMS-based local oscillator (LO). The NEMS resonator is tuned every 0.4 seconds during the 4 s transmission (a total of 10 times).
